# An Overview of the Spindle Assembly Checkpoint Status in Oral Cancer

**DOI:** 10.1155/2014/145289

**Published:** 2014-06-03

**Authors:** José Henrique Teixeira, Patrícia Manuela Silva, Rita Margarida Reis, Inês Moranguinho Moura, Sandra Marques, Joana Fonseca, Luís Silva Monteiro, Hassan Bousbaa

**Affiliations:** ^1^CESPU, Instituto de Investigação e Formação Avançada em Ciências e Tecnologias da Saúde, Rua Central de Gandra 1317, 4585-116 Gandra, Portugal; ^2^Centre for Molecular and Structural Biomedicine, CBME/IBB, University of Algarve, 8005-139 Faro, Portugal; ^3^Centro de Química Medicinal da Universidade do Porto (CEQUIMED-UP), Rua de Jorge Viterbo Ferreira 228, 4050-313 Porto, Portugal; ^4^Centro Interdisciplinar de Investigação Marinha e Ambiental (CIIMAR/CIMAR), Universidade do Porto, Rua dos Bragas 289, 4050-123 Porto, Portugal

## Abstract

Abnormal chromosome number, or aneuploidy, is a common feature of human solid tumors, including oral cancer. Deregulated spindle assembly checkpoint (SAC) is thought as one of the mechanisms that drive aneuploidy. In normal cells, SAC prevents anaphase onset until all chromosomes are correctly aligned at the metaphase plate thereby ensuring genomic stability. Significantly, the activity of this checkpoint is compromised in many cancers. While mutations are rather rare, many tumors show altered expression levels of SAC components. Genomic alterations such as aneuploidy indicate a high risk of oral cancer and cancer-related mortality, and the molecular basis of these alterations is largely unknown. Yet, our knowledge on the status of SAC components in oral cancer remains sparse. In this review, we address the state of our knowledge regarding the SAC defects and the underlying molecular mechanisms in oral cancer, and discuss their therapeutic relevance, focusing our analysis on the core components of SAC and its target Cdc20.

## 1. Introduction

Oral cancer is amongst the most common malignancy affecting mainly individuals with a history of tobacco and alcohol abuse [1]. Significantly, overall oral cancer-related mortality remained unchanged over the past two decades [2] despite significant improvement in quality of life thanks to advances in surgical techniques, radiotherapy, and chemotherapy. Thus, a major challenge in oral cancer diagnosis and treatment is to identify new therapeutic targets.

Contrary to microsatellite instability (MIN) which is rather rare, chromosomal instability (CIN) is the most frequent form of genetic instability in oral cancer, with frequent gains and losses of whole chromosomes or chromosomal segments [3]. CIN status in oral cancer was associated with a poor prognosis [3, 4]. Therefore, understanding the molecular mechanisms that underlie CIN is relevant to the clinic as it may lead to new anticancer therapy or to the discovery of new and more accurate prognostic markers that help guide therapeutic choices.

A number of reports demonstrated that alterations in the spindle assembly checkpoint (SAC) components can generate aneuploidy and induce tumor formation in animal models [5–7]. The SAC is a highly conserved surveillance mechanism that functions during mitosis to ensure accurate chromosome distribution between the two daughter cells [8, 9]. At the onset of mitosis, SAC proteins assemble on unattached kinetochores to produce an inhibitory signal that prevents the onset of anaphase until all chromosomes became bipolarly attached to microtubules of the mitotic spindle. It is thus expectable that alterations in SAC protein levels will produce abnormal chromosome segregation thus generating aneuploidy.

Given the unstable karyotype found in oral cancer cells and the role that SAC has in the generation of aneuploidy, this review aimed at presenting the state of our knowledge on SAC dysfunctions in oral cancer and discussing its therapeutic potential.

## 2. Oral Cancer

Oral cancer constitutes a major health problem being the sixth most common human cancer worldwide, with an incidence of more than 300,000 cases annually, with variations between countries and geographical areas [10]. In Southeast Asia, oral cancer shows the highest incidence and prevalence due to influence of tobacco and betel quid chewing habits [11]. High-risk human papilloma viruses (HR-HPVs) and Epstein-Barr virus (EBV) have also been identified as increasingly important risk factors [12–16]. Within the spectrum of oral malignancies, almost 90 percent are squamous cell carcinomas (SCC) [17]. Despite advances in knowledge on prevention and treatment of oral cancer, a low survival rate (near 50%) has been observed during the last decades [2, 18]. This is probably related to the late diagnosis, at a time when cancer has already metastasized. Indeed, patients have a better prognosis if they are treated in early stages, with a 5-year survival rate as high as 80% [12, 19, 20]. Suitable markers to early detect oral cancer and also to differentiate patients with more risk for recurrence or disease progression are therefore urgently needed [21].

Several genetic alterations including mutations, amplifications, translocations, or methylations have been implicated in oral cancer progression, affecting several tumor suppressors such as TP53 and RB and oncogenes like cyclins, EGFR, and* ras *[11, 22]. Through these alterations, tumor cells acquire autonomous growth and evade growth-inhibitory signals, leading to uncontrolled tumor growth. Mutations in TP53 gene are the most common genetic alterations, affecting around 45% of oral carcinomas [23, 24] and are associated with smoking [10]. These mutations affect DNA repair and compromise DNA damage-induced apoptosis, resulting in further genetic abnormalities and malignancy [10]. Expression of upstream regulators of pRb function, such as TP16, is also commonly altered [25] and may be involved in early stages of oral tumorigenesis [26]. In addition to overexpression of the* ras *oncogene family, which is linked to malignant transformation [27], EGFR, the receptor of EGF and TGF-*α*, is also commonly overexpressed in oral cancers. EGFR overexpression is linked to differentiation and aggressiveness of the tumors [26]. Also, overexpression of Cyclin D1 is frequent and is associated with recurrence and nodal metastasis [23].

Despite the advances in our understanding of the molecular basis of oral carcinogenesis and of oral cancer therapy, detailed information on the mechanisms that drive stepwise tumor progression is still missing. Such information may help uncover new biomarkers and develop potential therapeutic targets.

A common hallmark of several human cancers, including oral cancer, is aneuploidy, mainly defined as abnormal number of chromosomes. Mechanistically, chromosome missegregation due to abnormal mitosis is thought as one of the driving forces toward aneuploidy. Accurate chromosome segregation during mitosis is monitored by the spindle assembly checkpoint, a signaling pathway that inhibits anaphase onset until all chromosomes are aligned at the metaphase equator [28]. Defects in this checkpoint are common in many cancers and are associated with aneuploidy generation and tumor progression.

## 3. Spindle Assembly Checkpoint

**Figure 1:**
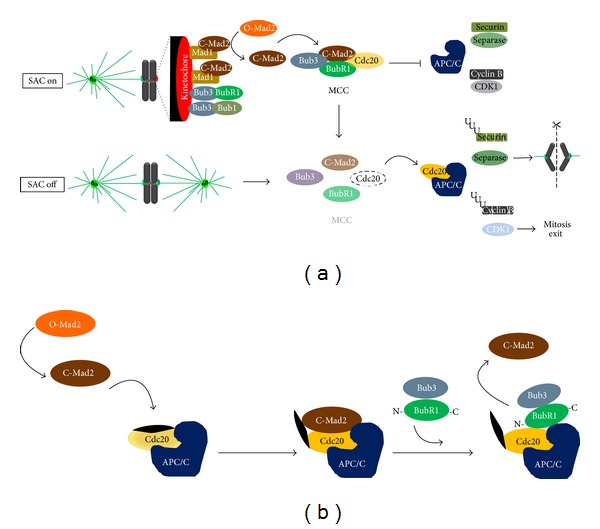
Current models of the signaling pathway of the spindle assembly checkpoint (SAC). (a) According to the first model, the presence of unattached or inappropriate attached kinetochores activates the SAC (SAC on). At the kinetochore level, the complex Mad1-“closed” Mad2 generates a diffusible signal, converting the cytosolic “Open” Mad2 into new “Closed” Mad2, which in association with Bub3, BubR1, and Cdc20 forms the mitotic checkpoint complex. Mitotic checkpoint complex (MCC) sequesters Cdc20 preventing activation of the anaphase promoting complex/cyclosome (APC/C). Once all chromosomes are properly attached, MCC disassembles (SAC off) and Cdc20 is free to activate the APC/C that targets Securin and Cyclin B for degradation. This way, Separase is released and cleaves Coesins allowing sister chromatid separation, while Cyclin B degradation allows mitosis exit. (b) According to the second model, cytosolic “closed” Mad2 promotes a conformational change in Cdc20, allowing its binding to the N terminus of BubR1 bound to Bub3, which maintain the APC/C inhibited thus preventing anaphase onset. Once this complex is formed, “closed” Mad2 is released and returns to cytosol.

Error-free chromosome segregation depends on the successful attachment of chromosomes, through its sister kinetochores, to microtubules from the mitotic spindle [29]. By monitoring the nature of kinetochore-microtubules interactions, the spindle assembly checkpoint (SAC), a surveillance and error-sensitive mechanism, avoids a premature sister-chromatid separation and ensures genomic stability [8]. The key signal for SAC activation is the presence of unattached or improperly attached kinetochores, which acts as a catalytic platform for the assembly of the mitotic checkpoint complex (MCC), an inhibitory signal of the metaphase to anaphase transition. The MCC comprises the highly conserved proteins Mad2, BubR1, and Bub3 in association with Cdc20 protein, a coactivator of the E3 ubiquitin ligase anaphase promoting complex/cyclosome (APC/C) [30]. Once this complex is generated, Cdc20 is unable to activate the APC/C, preventing anaphase onset by inhibiting Securin and Cyclin B degradation by ubiquitin/proteasome system (Figure 1(a)). A single free-kinetochore is able to activate the SAC transduction pathway and sustain a mitotic arrest [31]. This is possible because the inhibitory signal that emanates from the unattached kinetochore diffuses into and is further amplified within the cytoplasm. Such diffusion and amplification of the inhibitory signal are based on catalytic conformational conversion of Mad2 protein. At unattached kinetochores, the binary complex Mad1-“closed” Mad2 acts as a scaffold for a continuous conversion of the cytosolic “open” Mad2 into “closed” Mad2, which is able to bind Cdc20 and inhibit APC/C activity [32–35]. Recently, it was reported that a “closed” Mad2-dependent Cdc20 conformational change allows the binding of Cdc20 with the N terminus of BubR1 bound to Bub3, which in turn inhibits the APC/C activity and consequently anaphase onset (Figure 1(b)) [36]. Additional SAC components include the kinases Bub1, monopolar spindle 1 (Mps1), and Aurora B which are required for effective checkpoint signaling. Bub1, which forms a constitutive complex with Bub3 [37], is required for kinetochore recruitment of BubR1, Mad1, and Mad2 [38]. This kinase also contributes to APC/C inhibition through Cdc20-phosphorylation [39, 40]. In vertebrate cells, kinetochore targeting of Mad1 and Mad2 is also dependent on Mps1 which in turn is required for Aurora B kinase activation [41–44]. Aurora B is a component of chromosomal passenger complex (CPC), which also includes INCENP, Survivin, and Borealin, and has a role in correcting aberrant kinetochore-microtubule attachments [45]. In a process that involves substrate phosphorylation, Aurora B senses and destabilizes improper kinetochore-microtubule attachments, giving a second chance for successful chromosomes biorientation and alignment, thereby avoiding an aneuploid end [9, 46, 47]. Besides its role in error-correction, Aurora B also plays an important role in BubR1 and Mad2 kinetochore recruitment [48]. Once all chromosomes align at the metaphase plate, with proper bipolar attachments, the “wait anaphase” inhibitory signal must be extinguished, a process known as SAC silencing, to allow mitosis progression. A predominant mechanism of SAC silencing implies disassembly of existing MCC and preventing assembly of new MCC [49]. Once MCC is extinguished, Cdc20 becomes free to activate the APC/C which targets Securin and Cyclin B for degradation, thus leading to sister-chromatid separation and mitotic exit.

## 4. Spindle Assembly Checkpoint and Aneuploidy

Theodor Boveri has suggested that aneuploid progeny, resulting from disrupted mitosis, becomes the precursor cells of tumors [50]. In fact, nowadays it is well known that aneuploidy is a common feature of solid human tumors and is a contributor factor in tumorigenesis [28]. In order to maintain the same karyotype, every cell cycle, the cell must ensure that each daughter cell only receives one copy of each chromosome. Since this process is controlled by SAC, a compromised SAC will result in aneuploid cells, with too many or too few chromosomes, a state that contributes to carcinogenesis [51]. Actually, there are evidences that a weak mitotic checkpoint correlates with an increase in aneuploid cells. Although impaired, this weak SAC is not null, as cells are able to divide and survive. However, the gain or loss of whole chromosomes is frequent in these cells, ultimately leading to aneuploidy [50]. Chromosome gain or loss is known as chromosomal instability (CIN), the basis of aneuploidy and cancer. In fact, most cancer cells exhibit CIN and, frequently, high rate of aneuploidy and CIN is correlated with a poor patient prognosis [52, 53].

From a therapeutic point of view, chronic spindle assembly checkpoint activation is commonly used in chemotherapy and relies on the use of microtubule-targeting agents, which by disrupting microtubule dynamics elicits a long-term SAC response that frequently ends in mitotic cell death [54]. However, some cells are able to escape cell death and exit mitosis, a mechanism coined mitotic slippage [55]. Cell fate after SAC-dependent mitotic arrest induced by antimicrotubule drugs was suggested to be determined by two independent processes that run in parallel: (i) if the apoptotic machinery is efficiently activated, during the mitotic arrest, before Cyclin B reaches a threshold of degradation, then the cell is committed to die; (ii) if the threshold of Cyclin B degradation is reached before efficient activation of apoptosis, then the cell will exit without dying in mitosis [56]. Paradoxically, complete SAC inactivation also results in cell death. This potential anticancer strategy relies on the fact that, without a functional SAC, CIN is increased to a rate that produces massive missegregations resulting in unviable progeny. Taken together, this suggests that a careful fine-tuning of SAC activity is required for cell survival, since a weakened SAC can favor tumorigenesis but the absence or chronic activation of SAC results in apoptosis, even in tumor cells [52].

## 5. Spindle Assembly Checkpoint and Oral Squamous Cell Carcinoma

Oral squamous cell carcinoma (OSCC), like most other cancers, exhibits aneuploid cells. This carcinoma is characterized by complex karyotypes, often near-triploid, and contains multiple structural and numeric genetic abnormalities [57]. As stated above, defects in SAC are amongst the causes of aneuploidy in various cancers. As for oral cancer, there are only few reports that described SAC alterations in OSCC (Table 1). Overexpression of Cdc20, at mRNA levels, was reported in both OSCC-derived cell lines and primary head and neck squamous cell carcinoma (HNSCC) tissues [58]. Cell lines overexpressing Cdc20 undergo mitosis, with decreased Cyclin B levels, even in the presence of the microtubule disruption drug nocodazole, demonstrating an impairment of SAC function [58]. Cdc20 overexpression was also observed in OSCC-derived cell lines with p53 and p16 defects [59]. The same study reported Mad2 downregulation in OSCC cell lines, which may account for the complex karyotypes in these cells [58]. Interestingly, downregulated Mad2 and BubR1 increased sensitivity of esophageal squamous cell carcinoma lines to antimicrotubule agents currently used in chemotherapy, highlighting the predictive value of these SAC proteins for anticancer drug sensitivity [60]. Recently, Cdc20 overexpression in OSCC histological samples was linked to a poor prognosis, suggesting Cdc20 as a novel independent prognostic factor, as well as a molecular marker to categorize high-risk OSCC subgroups [61]. BubR1 was found to be overexpressed in OSCC samples and, in less extent, in oral potentially malignant disorders, comparatively to normal oral mucosa, suggesting that BubR1 upregulation is an early event in oral carcinogenesis pathway [62]. High BubR1 expression was associated with shorter survival in oral malignant lesions and, interestingly, a possible correlation between HPV infection and BubR1 overexpression was suggested [63]. In contrast, another study shows that overexpression of BubR1 was associated with a less advanced tumor stage, but patients with overexpression of BubR1 showed shorter recurrence-free survival than those without it, making BubR1 a promising prognostic marker in patients with OSCC [64]. Curiously, the proliferation marker Ki-67 did not demonstrate a statistical significant correlation with BubR1 expression, contrasting with a previous report in patients with tonsillar carcinomas [65]. Another SAC protein that was also found to be overexpressed in oral carcinoma is the Aurora B kinase. A positive correlation between the clinicopathological parameters, disease-free survival and Aurora B expression, was reported, suggesting that Aurora B status might be useful to identify the risk patients and can be used as a potential prognostic factor [66]. Aurora B was involved in lymph node metastatic process and poor differentiation grade. Together with its colocalization pattern with Ki-67, a role of Aurora B in tumor progression was suggested [67]. Recently, overexpression of Aurora B was associated with advanced tumor stage and a poor prognosis of OSCC patients [68], strengthening the potential of Aurora B status as an OSCC predictive biomarker [66]. Overall, the overexpression patterns of Cdc20, BubR1, and Aurora B proteins in OSCC (Table 1) are similar to those observed in other tumor types [69–71].

We have focused our analysis on core SAC components (Mad1, Mad2, Bub1, BubR1, Bub3, Aurora B, and Mps1) as well as on the main target of SAC (Cdc20). As described above and summarized in Table 1, little information exists as to the expression status of SAC proteins in oral cancer comparatively to other cancers. Overall expression status of Mad2, BubR1, Aurora B, and Cdc20 in oral cancer seems concordant between different reports, with a general tendency to upregulation, except for Mad2. However, there is no report as to the expression status of the other aforementioned core SAC proteins. This missing information is crucial as SAC activity, and the significance of its alteration to cancer, depends on the fine-tuned equilibrium between all of its components and their expression levels.

## 6. Conclusion

The SAC is compromised in many malignant tumors and has been implicated as a contributor to aneuploidy and carcinogenesis in animals and human. Over- and underexpression of SAC components were reported in various types of cancer. In many cases, the SAC component expression status is linked with a high proliferation activity and a poor prognosis. Consequently, targeting the SAC pathway for anticancer therapies has been investigated [72] and some drugs are currently in various stages of clinical trials. For instance, Aurora B kinase inhibitors induce apoptosis* in vitro* and* in vivo* and are being tested in clinical trials [73–75]. However, and as it can be concluded from this review, there is still a lack of knowledge on the status of SAC components in oral cancer. Extending our understanding of the expression profile to all SAC components, and to other components participating in the SAC, may be useful to oral cancer treatment. Eventually, this would help determine whether oral cancer patients can potentially benefit from anti-SAC agents that are currently in active clinical development.

## Figures and Tables

**Table 1:** Expression levels of core spindle assembly checkpoint components in oral squamous cell carcinoma.

SAC protein	Samples	Expression levels	References
Cdc20	OSCC cell lines (7 out of 10) Primary HNSCC (7 out of 10 cases)	Overexpression	[58]
	OSCC cell lines (2 of 7)	Overexpression	[59]
	OSCC (37 out of 65 cases)	Overexpression	[61]

Mad2	OSCC cell lines (7 out of 7)	Underexpression	[59]

BubR1	OSCC (43 out of 43 cases) Oral PMD (75 out of 77 cases)	Overexpression	[62]
	OSCC^IS^ (15 out of 20 cases) OSCC^WT ^(19 out of 27 cases) OSCC^W ^(18 out of 23 cases) Cervical LN (15 out of 23 cases)	Overexpression	[63]
	OL^NM^ (16 out of 16 cases)	Underexpression	
	OSCC (11 out of 49 cases)	Overexpression	[64]

Aurora B	OSCC cell lines (7 out of 7) OSCC (71 out of 101)	Overexpression	[66]
	OSCC (40 out of 40 cases)	Overexpression	[67]
	OSCC (162 out of 215 cases)	Overexpression	[68]

PMD: potentially malignant disorders; OSCC^IS^: *in situ* OSCC; OSCC^WT^: invasive OSCC without metastasis; OSCC^W^: invasive OSCC with metastasis; cervical LN: lymph nodes; OL^NM^: nonmalignant oral lesions.

## References

[1] Forastiere A, Koch W, Trotti A, Sidransky D (2001). Head and neck cancer. *The New England Journal of Medicine*.

[2] Warnakulasuriya S (2009). Global epidemiology of oral and oropharyngeal cancer. *Oral Oncology*.

[3] Reshmi SC, Gollin SM (2005). Chromosomal instability in oral cancer cells. *Journal of Dental Research*.

[4] Sato H, Uzawa N, Takahashi K-I, Myo K, Ohyama Y, Amagasa T (2010). Prognostic utility of chromosomal instability detected by fluorescence in situ hybridization in fine-needle aspirates from oral squamous cell carcinomas. *BMC Cancer*.

[5] Diaz-Rodríguez E, Sotillo R, Schvartzman J-M, Benezra R (2008). Hec1 overexpression hyperactivates the mitotic checkpoint and induces tumor formation in vivo. *Proceedings of the National Academy of Sciences of the United States of America*.

[6] Sotillo R, Hernando E, Díaz-Rodríguez E (2007). Mad2 overexpression promotes aneuploidy and tumorigenesis in mice. *Cancer Cell*.

[7] Weaver BAA, Silk AD, Montagna C, Verdier-Pinard P, Cleveland DW (2007). Aneuploidy acts both oncogenically and as a tumor suppressor. *Cancer Cell*.

[8] Musacchio A, Salmon ED (2007). The spindle-assembly checkpoint in space and time. *Nature Reviews Molecular Cell Biology*.

[9] Silva P, Barbosa J, Nascimento AV, Faria J, Reis R, Bousbaa H (2011). Monitoring the fidelity of mitotic chromosome segregation by the spindle assembly checkpoint. *Cell Proliferation*.

[10] Tanaka T, Tanaka M, Tanaka T (2011). Oral carcinogenesis and oral cancer chemoprevention: a review. *Pathology Research International*.

[11] Tsantoulis PK, Kastrinakis NG, Tourvas AD, Laskaris G, Gorgoulis VG (2007). Advances in the biology of oral cancer. *Oral Oncology*.

[12] Yan W, Wistuba II, Emmert-Buck MR, Erickson HS (2011). Squamous cell carcinoma—similarities and differences among anatomical sites. *American Journal of Cancer Research*.

[13] Rothenberg SM, Ellisen LW (2012). The molecular pathogenesis of head and neck squamous cell carcinoma. *Journal of Clinical Investigation*.

[14] Llewellyn CD, Johnson NW, Warnakulasuriya KAAS (2001). Risk factors for squamous cell carcinoma of the oral cavity in young people—a comprehensive literature review. *Oral Oncology*.

[15] Miller CS, White DK (1996). Human papillomavirus expression in oral mucosa, premalignant conditions, and squamous cell carcinoma: a retrospective review of the literature. *Oral Surgery, Oral Medicine, Oral Pathology, Oral Radiology, and Endodontics*.

[16] Sugerman PB, Shillitoe EJ (1997). The high risk human papillomaviruses and oral cancer: evidence for and against a causal relationship. *Oral Diseases*.

[17] Monteiro LS, Antunes L, Bento MJ, Warnakulasuriya S (2013). Incidence rates and trends of lip, oral and oro-pharyngeal cancers in Portugal. *Journal of Oral Pathology & Medicine*.

[18] Neville BW, Day TA (2002). Oral cancer and precancerous lesions. *Ca: A Cancer Journal for Clinicians*.

[19] Mehrotra R, Gupta A, Singh M, Ibrahim R (2006). Application of cytology and molecular biology in diagnosing premalignant or malignant oral lesions. *Molecular Cancer*.

[20] Patel V, Leethanakul C, Gutkind JS (2001). New approaches to the understanding of the molecular basis of oral cancer. *Critical Reviews in Oral Biology and Medicine*.

[21] Islam MN, Kornberg L, Veenker E, Cohen DM, Bhattacharyya I (2010). Anatomic site based ploidy analysis of oral premalignant lesions. *Head and Neck Pathology*.

[22] Scully C, Bedi R (2000). Ethnicity and oral cancer. *The Lancet Oncology*.

[23] Gleich LL, Salamone FN (2002). Molecular genetics of head and neck cancer. *Cancer Control*.

[24] Humayun S, Prasad VR (2011). Expression of p53 protein and ki-67 antigen in oral premalignant lesions and oral squamous cell carcinomas: an immunohistochemical study. *National Journal of Maxillofacial Surgery*.

[25] Liggett WH, Sidransky D (1998). Role of the p16 tumor suppressor gene in cancer. *Journal of Clinical Oncology*.

[26] Williams HK (2000). Molecular pathogenesis of oral squamous carcinoma. *Journal of Clinical Pathology: Molecular Pathology*.

[27] Yarbrough WG, Shores C, Witsell DL, Weissler MC, Fidler ME, Gilmer TM (1994). *ras* mutations and expression in head and neck squamous cell carcinomas. *Laryngoscope*.

[28] Holland AJ, Cleveland DW (2009). Boveri revisited: chromosomal instability, aneuploidy and tumorigenesis. *Nature Reviews Molecular Cell Biology*.

[29] Foley EA, Kapoor TM (2013). Microtubule attachment and spindle assembly checkpoint signalling at the kinetochore. *Nature Reviews Molecular Cell Biology*.

[30] Sudakin V, Chan GKT, Yen TJ (2001). Checkpoint inhibition of the APC/C in HeLa cells is mediated by a complex of BUBR1, BUB3, CDC20, and MAD2. *The Journal of Cell Biology*.

[31] Rieder CL, Cole RW, Khodjakov A, Sluder G (1995). The checkpoint delaying anaphase in response to chromosome monoorientation is mediated by an inhibitory signal produced by unattached kinetochores. *The Journal of Cell Biology*.

[32] de Antoni A, Pearson CG, Cimini D (2005). The Mad1/Mad2 complex as a template for Mad2 activation in the spindle assembly checkpoint. *Current Biology*.

[33] Yu H (2006). Structural activation of Mad2 in the mitotic spindle checkpoint: The two-state Mad2 model versus the Mad2 template model. *The Journal of Cell Biology*.

[34] Luo X, Yu H (2008). Protein metamorphosis: the two-state behavior of Mad2. *Structure*.

[35] Skinner JJ, Wood S, Shorter J, Englander SW, Black BE (2008). The Mad2 partial unfolding model: regulating mitosis through Mad2 conformational switching. *The Journal of Cell Biology*.

[36] Han J, Holland A, Fachinetti D, Kulukian A, Cetin B, Cleveland D (2013). Catalytic assembly of the mitotic checkpoint inhibitor BubR1-Cdc20 by a Mad2-induced functional switch in Cdc20. *Molecular Cell*.

[37] Brady DM, Hardwick KG (2000). Complex formation between Mad1p, Bub1p and Bub3p is crucial for spindle checkpoint function. *Current Biology*.

[38] Johnson VL, Scott MIF, Holt SV, Hussein D, Taylor SS (2004). Bub1 is required for kinetochore localization of BubR1, Cenp-E, Cenp-F and Mad2, and chromosome congression. *Journal of Cell Science*.

[39] Tang Z, Shu H, Oncel D, Chen S, Yu H (2004). Phosphorylation of Cdc20 by Bub1 provides a catalytic mechanism for APC/C inhibition by the spindle checkpoint. *Molecular Cell*.

[40] Kang J, Yang M, Li B (2008). Structure and substrate recruitment of the human spindle checkpoint kinase Bub1. *Molecular Cell*.

[41] Abrieu A, Magnaghi-Jaulin L, Kahana JA (2001). Mps1 is a kinetochore-associated kinase essential for the vertebrate mitotic checkpoint. *Cell*.

[42] Jelluma N, Dansen TB, Sliedrecht T, Kwiatkowski NP, Kops GJPL (2010). Release of Mps1 from kinetochores is crucial for timely anaphase onset. *The Journal of Cell Biology*.

[43] Liu S-T, Chan GKT, Hittle JC, Fujii G, Lees E, Yen TJ (2003). Human MPS1 kinase is required for mitotic arrest induced by the loss of CENP-E from kinetochores. *Molecular Biology of the Cell*.

[44] Vigneron S, Prieto S, Bernis C, Labbé J-C, Castro A, Lorca T (2004). Kinetochore localization of spindle checkpoint proteins: who controls whom?. *Molecular Biology of the Cell*.

[45] Ruchaud S, Carmena M, Earnshaw WC (2007). The chromosomal passenger complex: one for all and all for one. *Cell*.

[46] Tanaka TU, Rachidi N, Janke C (2002). Evidence that the Ipl1-Sli15 (Aurora Kinase-INCENP) complex promotes chromosome bi-orientation by altering kinetochore-spindle pole connections. *Cell*.

[47] DeLuca KF, Lens SMA, DeLuca JG (2011). Temporal changes in Hec1 phosphorylation control kinetochore—microtubule attachment stability during mitosis. *Journal of Cell Science*.

[48] Ditchfield C, Johnson VL, Tighe A (2003). Aurora B couples chromosome alignment with anaphase by targeting BubR1, Mad2, and Cenp-E to kinetochores. *The Journal of Cell Biology*.

[49] Howell BJ, McEwen BF, Canman JC (2001). Cytoplasmic dynein/dynactin drives kinetochore protein transport to the spindle poles and has a role in mitotic spindle checkpoint inactivation. *The Journal of Cell Biology*.

[50] Kops GJPL, Weaver BAA, Cleveland DW (2005). On the road to cancer: aneuploidy and the mitotic checkpoint. *Nature Reviews Cancer*.

[51] Kops GJPL, Foltz DR, Cleveland DW (2004). Lethality to human cancer cells through massive chromosome loss by inhibition of the mitotic checkpoint. *Proceedings of the National Academy of Sciences of the United States of America*.

[52] Janssen A, Medema RH (2011). Mitosis as an anti-cancer target. *Oncogene*.

[53] Salmela A-L, Kallio MJ (2013). Mitosis as an anti-cancer drug target. *Chromosoma*.

[54] Chan K-S, Koh C-G, Li H-Y (2012). Mitosis-targeted anti-cancer therapies: where they stand. *Cell Death and Disease*.

[55] Brito DA, Rieder CL (2006). Mitotic checkpoint slippage in humans occurs via cyclin B destruction in the presence of an active checkpoint. *Current Biology*.

[56] Gascoigne KE, Taylor SS (2008). Cancer cells display profound intra- and interline variation following prolonged exposure to antimitotic drugs. *Cancer Cell*.

[57] Gollin SM (2001). Chromosomal alterations in squamous cell carcinomas of the head and neck: window to the biology of disease. *Head & Neck*.

[58] Mondal G, Sengupta S, Panda CK, Gollin SM, Saunders WS, Roychoudhury S (2007). Overexpression of Cdc20 leads to impairment of the spindle assembly checkpoint and aneuploidization in oral cancer. *Carcinogenesis*.

[59] Thirthagiri E, Robinson CM, Huntley S (2007). Spindle assembly checkpoint and centrosome abnormalities in oral cancer. *Cancer Letters*.

[60] Tanaka K, Mohri Y, Ohi M (2008). Mitotic checkpoint genes, hsMAD2 and BubR1, in oesophageal squamous cancer cells and their association with 5-fluorouracil and cisplatin-based radiochemotherapy. *Clinical Oncology*.

[61] Moura IM, Delgado ML, Silva PM (2014). High CDC20 expression is associated with poor prognosis in oral squamous cell carcinoma. *Journal of Oral Pathology & Medicine*.

[62] Hsieh P-C, Chen Y-K, Tsai K-B (2010). Expression of BUBR1 in human oral potentially malignant disorders and squamous cell carcinoma. *Oral Surgery, Oral Medicine, Oral Pathology, Oral Radiology and Endodontology*.

[63] Lira RCP, Miranda FA, Guimarães MCM (2010). BUBR1 expression in benign oral lesions and squamous cell carcinomas: correlation with human papillomavirus. *Oncology Reports*.

[64] Rizzardi C, Torelli L, Barresi E (2011). BUBR1 expression in oral squamous cell carcinoma and its relationship to tumor stage and survival. *Head & Neck*.

[65] Hannisdal K, Burum-Auensen E, Schjølberg A, de Angelis PM, Clausen OPF (2010). Correlation between reduced expression of the spindle checkpoint protein BubR1 and bad prognosis in tonsillar carcinomas. *Head & Neck*.

[66] Pannone G, Hindi SAH, Santoro A (2011). Aurora B expression as a prognostic indicator and possibile therapeutic target in oral squamous cell carcinoma. *International Journal of Immunopathology and Pharmacology*.

[67] Qi G, Ogawa I, Kudo Y (2007). Aurora-B expression and its correlation with cell proliferation and metastasis in oral cancer. *Virchows Archiv*.

[68] Chen J-H, Yeh K-T, Yang Y-M, Chang J-G, Lee H-E, Hung S-Y (2013). High expressions of histone methylation- and phosphorylation-related proteins are associated with prognosis of oral squamous cell carcinoma in male population of Taiwan. *Medical Oncology*.

[69] Wu W-J, Hu K-S, Wang D-S (2013). CDC20 overexpression predicts a poor prognosis for patients with colorectal cancer. *Journal of Translational Medicine*.

[70] Yamamoto Y, Matsuyama H, Chochi Y (2007). Overexpression of BUBR1 is associated with chromosomal instability in bladder cancer. *Cancer Genetics and Cytogenetics*.

[71] Lin Z-Z, Jeng Y-M, Hu F-C (2010). Significance of Aurora B overexpression in hepatocellular carcinoma. Aurora B overexpression in HCC. *BMC Cancer*.

[72] Schvartzman J-M, Sotillo R, Benezra R (2010). Mitotic chromosomal instability and cancer: mouse modelling of the human disease. *Nature Reviews Cancer*.

[73] Kollareddy M, Zheleva D, Dzubak P, Brahmkshatriya PS, Lepsik M, Hajduch M (2012). Aurora kinase inhibitors: progress towards the clinic. *Investigational New Drugs*.

[74] Dennis M, Davies M, Oliver S, D’Souza R, Pike L, Stockman P (2012). Phase i study of the Aurora B kinase inhibitor barasertib (AZD1152) to assess the pharmacokinetics, metabolism and excretion in patients with acute myeloid leukemia. *Cancer Chemotherapy and Pharmacology*.

[75] Wilkinson RW, Odedra R, Heaton SP (2007). AZD1152, a selective inhibitor of Aurora B kinase, inhibits human tumor xenograft growth by inducing apoptosis. *Clinical Cancer Research*.

